# Performance of Li_4_SiO_4_ Material for CO_2_ Capture: A Review

**DOI:** 10.3390/ijms20040928

**Published:** 2019-02-20

**Authors:** Xianyao Yan, Yingjie Li, Xiaotong Ma, Jianli Zhao, Zeyan Wang

**Affiliations:** 1 School of Energy and Power Engineering, Shandong University, Jinan 250061, China; yanxy1009@163.com (X.Y.); xiaotong.ma.mail@gmail.com (X.M.); sdzhaojl@sdu.edu.cn (J.Z.); 2 State Key Laboratory of Crystal Materials, Shandong University, Jinan 250100, China; wangzeyan@sdu.edu.cn

**Keywords:** CO_2_ capture, energy production process, Li_4_SiO_4_ material, modification

## Abstract

Lithium silicate (Li_4_SiO_4_) material can be applied for CO_2_ capture in energy production processes, such as hydrogen plants, based on sorption-enhanced reforming and fossil fuel-fired power plants, which has attracted research interests of many researchers. However, CO_2_ absorption performance of Li_4_SiO_4_ material prepared by the traditional solid-state reaction method is unsatisfactory during the absorption/regeneration cycles. Improving CO_2_ absorption capacity and cyclic stability of Li_4_SiO_4_ material is a research highlight during the energy production processes. The state-of-the-art kinetic and quantum mechanical studies on the preparation and CO_2_ absorption process of Li_4_SiO_4_ material are summarized, and the recent studies on the effects of preparation methods, dopants, and operating conditions on CO_2_ absorption performance of Li_4_SiO_4_ material are reviewed. Additionally, potential research thoughts and trends are also suggested.

## 1. Introduction

The emission of anthropogenic CO_2_ into the environment has aggravated the trend of global warming [1], which has become one of the most threatening problems in recent decades, and the largest emission sources of CO_2_ are fossil fuel-fired power plants [2]. Hence, various techniques have been reported to capture CO_2_ from the flue gas released from fossil fuel-fired power plants [3], and CO_2_ capture and storage (CCS) has been recognized as one of the most effective techniques to mitigate CO_2_ emission [4,5]. In the process of CCS, CO_2_ is captured from flue gas and stored for utilization and sequestration instead of being released to the environment directly. Recent studies have found that various lithium-based materials, such as LiFeO_2_ [6], Li_2_CuO_2_ [7], Li_2_ZrO_3_ [7,8,9], Li_8_SiO_6_ [10,11], and Li_4_SiO_4_ [9], are capable of effective CO_2_ capture. Among these materials, Li_4_SiO_4_, with a variety of applications [12,13], has better application potential, owing to its higher CO_2_ sorption capacity, cyclic stability than LiFeO_2_, Li_2_CuO_2_, and Li_8_SiO_6_, and lower cost than that of Li_2_ZrO_3_ [9]. Additionally, the regeneration temperature of Li_4_SiO_4_ material is much lower compared with the calcium-based CO_2_ sorbents, indicating that lower energy consumption is required for the regeneration. Li_4_SiO_4_ material is usually obtained by the solid-state reaction method with Li_2_CO_3_ and SiO_2_ at high temperature, which is shown in Equation (1) [14]:

(1) $$2Li_{2}CO_{3}+SiO_{2}↔Li_{4}SiO_{4}+2CO_{2}$$

**Figure 1:**
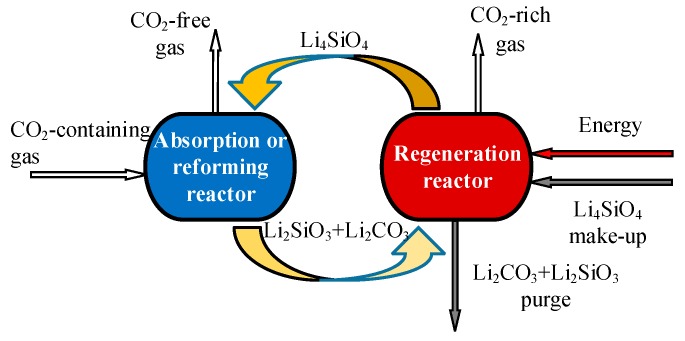
Application of Li_4_SiO_4_ material for CO_2_ absorption for fossil fuel-fired power plants or hydrogen plants based on sorption-enhanced reforming.

The basic reversible reaction for CO_2_ sorption by Li_4_SiO_4_ material follows Equation (2), and the process is shown in Figure 1. In the absorption reactor, CO_2_ in flue gas from fossil fuel-fired power plants or syngas from hydrogen plants based on sorption-enhanced reforming is absorbed by Li_4_SiO_4_ at 500 to 600 °C, thus the gas, almost free of CO_2_, is exhausted from the reactor. The generated Li_2_SiO_3_ and Li_2_CO_3_ are transported to the regeneration reactor, where Li_4_SiO_4_ is regenerated at temperatures higher than 700 °C and sent to the absorption reactor for the next CO_2_ absorption cycle, and CO_2_-rich gas can be obtained in the regeneration reactor.

(2) $$Li_{4}SiO_{4}+CO_{2}↔Li_{2}SiO_{3}+Li_{2}CO_{3}$$

It can be calculated according to Equation (2) that the theoretical CO_2_ absorption capacity of Li_4_SiO_4_ is 367 mg CO_2_/g Li_4_SiO_4_, which is much higher than that of Li_2_ZrO_3_ (125 mg/g) [9].

Additionally, CO_2_ absorption by Li_4_SiO_4_ material can also contribute to sorption-enhanced hydrogen production, as shown in Figure 1, where methane and ethanol are usually selected [15]. In the process of methane or ethanol reforming, CO_2_ is a necessary while undesired product. With in situ CO_2_ absorption by Li_4_SiO_4_ material, the concentration of CO_2_ in syngas can be reduced, and the reaction equilibrium of reforming can be shifted to hydrogen production simultaneously, thus the CO_2_ absorption capacity of Li_4_SiO_4_ material is the key factor to determine the hydrogen production efficiency [16,17]. This section will be discussed in detail in Section 6.

However, the CO_2_ absorption capacity and cyclic stability of pristine Li_4_SiO_4_ material prepared by the solid-state reaction method is low, which is mainly due to the smooth surface of pristine Li_4_SiO_4_ particles generated at high temperature, thus the surface area and pore volume of Li_4_SiO_4_ material are low, and the reaction between CO_2_ and Li_4_SiO_4_ is limited [18]. Therefore, a large number of works have been conducted to improve the pore structure of Li_4_SiO_4_, such as the application of organic precursors, which is conducive to the formation of pores, and doping with eutectic salts, which is favorable for the decrease of CO_2_ diffusion resistance [19,20].

Since CO_2_ absorption by Li_4_SiO_4_ material was firstly reported, abundant studies have been revealed the reaction mechanism and improved the cyclic CO_2_ absorption performance [21]. This work introduces the latest research progress on CO_2_ absorption performance of Li_4_SiO_4_ material. In addition, thermodynamic and kinetic comprehension of the reaction between CO_2_ and Li_4_SiO_4_ are illustrated, and strategies to enhance the cyclic CO_2_ absorption performance of Li_4_SiO_4_ material are summarized. Additionally, applications of Li_4_SiO_4_ material in sorption-enhanced hydrogen production are reviewed, and studies on CO_2_ absorption by Li_4_SiO_4_ material at the molecular scale are also reviewed briefly. Finally, the major drawback that hinders the large-scale application of Li_4_SiO_4_ material for CO_2_ absorption is introduced.

## 2. Thermodynamics and Kinetics of CO_2_ Absorption by Li_4_SiO_4_

### 2.1. Reaction Model for Synthesis of Li_4_SiO_4_

**Figure 2:**
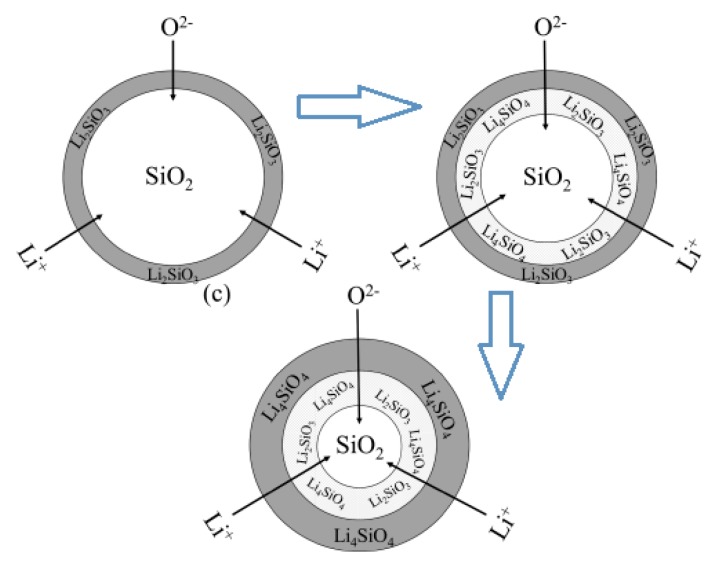
Core-shell model for synthesis of Li_4_SiO_4_ by the solid-state route [24].

Li_4_SiO_4_ material is usually synthesized by the solid-state reaction method, and the preparation process is illustrated by Equation (1) [22,23], and a core-shell model was suggested for the solid-state reaction between Li_2_CO_3_ and SiO_2_, which is shown in Figure 2. In the first step, Li_2_CO_3_ reacts with SiO_2_ at their contact part, and a thin Li_2_SiO_3_ layer is formed. Li_2_SiO_3_ is the intermediate product, which continues to react with Li_2_CO_3_ to form Li_4_SiO_4_ eventually. Li_4_SiO_4_ and Li_2_SiO_3_ layers become thicker with the reaction, and internal SiO_2_ is covered by the layers in the meantime. Thus, Li^+^ and O^2−^ must diffuse through the product layer before contacting with internal SiO_2_, and it is the limited step that limiting the synthesis of Li_4_SiO_4_, because the diffusion process is much slower than the reaction. Consequently, alternative synthesis methods and precursors for the synthesis of Li_4_SiO_4_ have been reported, which will be discussed in the following section [24].

### 2.2. Kinetic Study for CO_2_ Absorption by Li_4_SiO_4_

**Figure 3:**
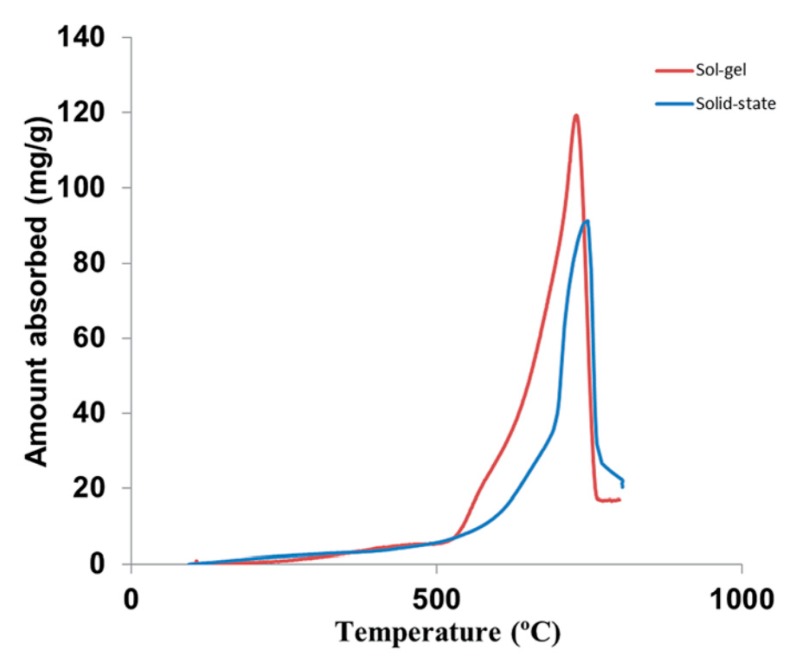
CO_2_ absorption by Li_4_SiO_4_ material in pure CO_2_ with respect to temperature [25].

Figure 3 shows the CO_2_ absorption performance of Li_4_SiO_4_ materials prepared by the solid-state reaction method and the sol-gel method in a thermogravimetric analyzer (TGA) [25], and the CO_2_ absorption amount (mg CO_2_/g sorbent) was used to evaluate the CO_2_ absorption capacity of Li_4_SiO_4_ materials, which is calculated according to Equation (3):

(3) $$C_{N}=\frac{m_{2}-m_{1}}{m_{1}}$$

where *C_N_* is the amount of CO_2_ absorbed by the Li_4_SiO_4_ material, mg/g; *N* represents the number of cycles; *m*_1_ represents the initial mass of Li_4_SiO_4_ material, g; and *m*_2_ represents the mass of the sample during CO_2_ absorption, mg. The CO_2_ absorption stage of Li_4_SiO_4_ occurs at temperatures lower than 400 °C, and the CO_2_ absorption rate increases suddenly when the temperature reaches around 500–600 °C. Weight losses of two Li_4_SiO_4_ materials are observed when the temperature exceeds 720 °C, indicating the reaction converts to the regeneration of Li_4_SiO_4_ materials, and the regeneration reaction is much faster than the absorption process. As shown in Figure 3, the CO_2_ absorption capacity of Li_4_SiO_4_ material prepared by the sol-gel method is higher than that prepared by the solid-state reaction method, which will be discussed in Section 3.3.3.

### 2.3. Thermodynamic Study for CO_2_ Capture by Li_4_SiO_4_

**Figure 4:**
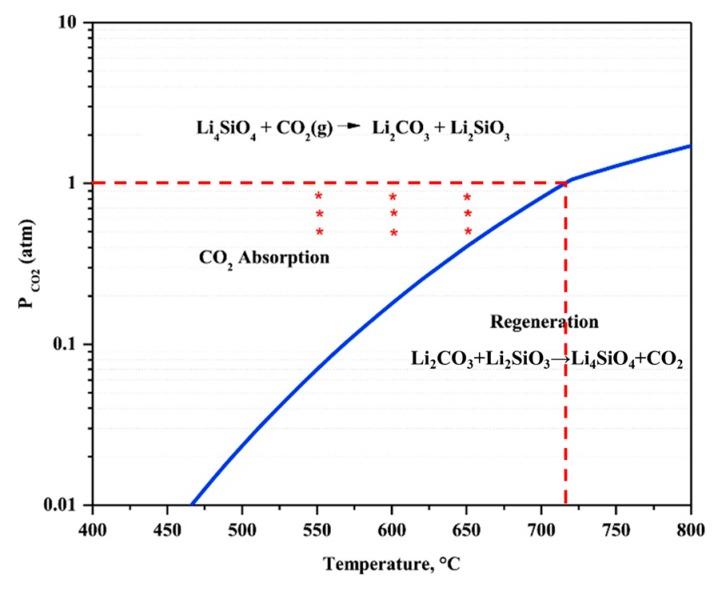
Equilibrium CO_2_ partial pressure as a function of temperature for the absorption of CO_2_ of Li_4_SiO_4_ [26].

Figure 4 shows the equilibrium partial pressure of CO_2_ over Li_4_SiO_4_ material as a function of temperature [26], and the maximum temperature of CO_2_ absorption by Li_4_SiO_4_ material is determined by the corresponding CO_2_ partial pressure. When CO_2_ partial pressure is 100% at 1 atm, it can be inferred from Figure 4 that the equilibrium temperature is around 715 °C, which agrees well with the results that discussed above. The corresponding temperature of CO_2_ absorption by Li_4_SiO_4_ material can be determined by the CO_2_ partial pressure, and CO_2_ absorption by Li_4_SiO_4_ material occurs when the temperature is lower than the equilibrium temperature, otherwise Li_4_SiO_4_ material is regenerated. As a result, CO_2_ absorption and regeneration regions of Li_4_SiO_4_ material are divided by the equilibrium line.

### 2.4. Reaction Mechanism and Reaction Model of CO_2_ Capture by Li_4_SiO_4_

**Figure 5:**
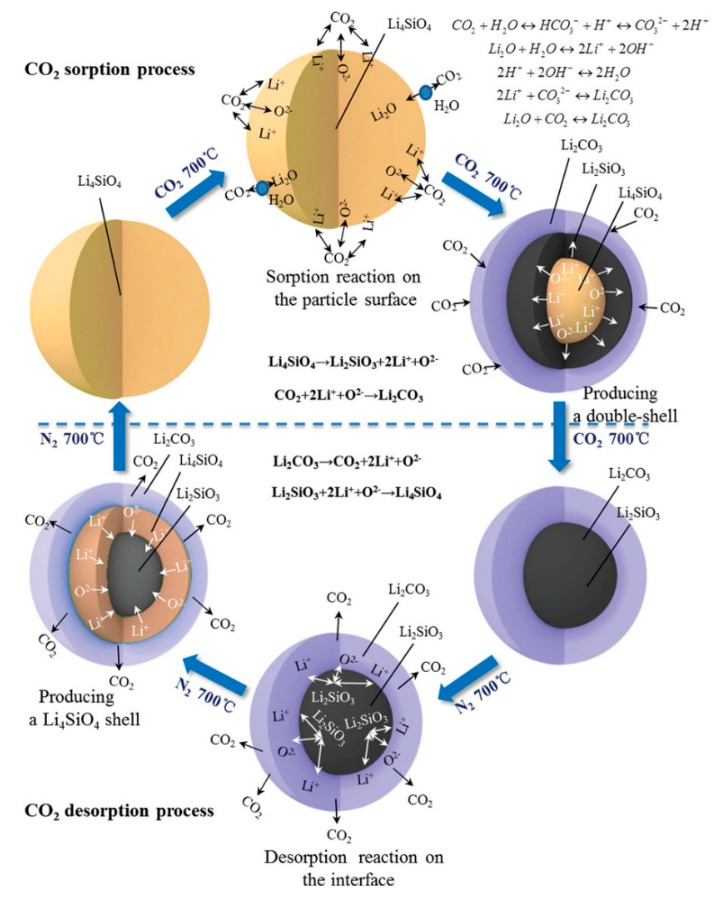
Double-shell mechanism of Li_4_SiO_4_ material for CO_2_ absorption and regeneration [27].

The double-shell mechanism is regarded as the most appropriate model for the reaction between CO_2_ and Li_4_SiO_4_ [27], which is schematically illustrated in Figure 5. At the beginning of the reaction, CO_2_ molecules react with Li_4_SiO_4_ particles to generate a double shell composed of Li_2_CO_3_ and Li_2_SiO_3_, which covers the internal Li_4_SiO_4_. Then the reactants diffuse through the double shell to continue the reaction, and the thickness of the double shell increases as the reaction proceeds. Thus, the second stage is much slower than the first stage, owing to the high diffusion resistance of the reactants. Therefore, decreasing the diffusion resistance is conducive to the reaction between CO_2_ and Li_4_SiO_4_. The presence of steam and doping of molten salts are believed to reduce the diffusion resistance in the double shell, which will be discussed in the following section. Additionally, the shrinking core model and the unreacted core model were well-reported in many studies, which are also involved with the external product shell and internal unreacted core, and the models are similar to that of the double-shell model.

**Figure 6:**
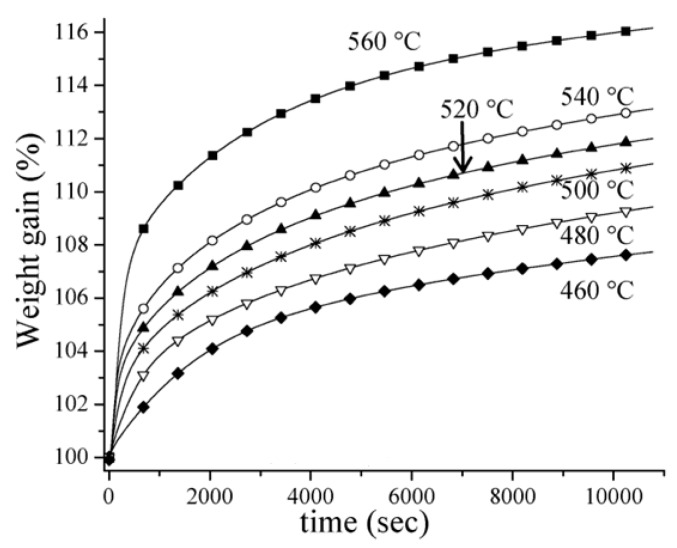
Isotherms obtained in a CO_2_ atmosphere at various temperatures [28].

TGA curves of CO_2_ absorption by Li_4_SiO_4_ at various temperatures are present in Figure 6, and weight gain of Li_4_SiO_4_ was used to evaluate its CO_2_ absorption performance, which can be calculated according to Equation (4):

(4) $$W_{N}=(C_{N}+1)\times 100\%$$

where *W_N_* is the weight gain of Li_4_SiO_4_ material during the *N*th cycles, wt.%; and *C_N_* is the amount of CO_2_ absorbed by Li_4_SiO_4_ material during the *N*th cycles, mg/g. CO_2_ absorption capacity of Li_4_SiO_4_ increases with the temperature rising from 460 to 560 °C. Li_4_SiO_4_ shows a fast CO_2_ absorption stage in a short time and a slow CO_2_ absorption stage in the following long time, which are controlled by the chemical reaction and diffusion, respectively [28]. Most of the TGA curves are fitted to the double exponential model, which is shown in Equation (5):

(5) $$y=A\exp^{-k_{1}t}+B\exp^{-k_{2}t}+C$$

where *y* represents the weight gain of Li_4_SiO_4_ material after CO_2_ absorption; *k*_1_ and *k*_2_ denote two exponential constants for the chemical reaction-controlled stage and the diffusion-controlled stage, respectively; and two pre-exponential factors *A* and *B* are the intervals that control the corresponding stages [28].

Table 1 presents the kinetic parameters of the double exponential model fitted to the reaction between CO_2_ and Li_4_SiO_4_ [28]. As presented in Table 1, the values of *k*_1_ are usually one order of magnitude higher than those of *k*_2_, and *B* are always larger than *A*, indicating that CO_2_ absorption over the surface of Li_4_SiO_4_ controlled by chemical reaction is a rapid process, and CO_2_ absorption controlled by diffusion occurs in a large interval of time. Thus, CO_2_ absorption controlled by diffusion is the limiting step hindering the absorption of CO_2_ by Li_4_SiO_4_ [29,30].

Although the double exponential model is widely used due to its simplicity, Ortiz et al. [26] thought that this model was short of the theoretical mechanism to support its fitting with the experimental data. Zhang et al. [27] reported that the Avrami–Erofeev model was relevant to the reaction mechanism of the formation and growth of product crystals, which are shown as Equations (6) and (7):

(6) $$d\alpha /dt=Kn(1-\alpha ){\left[-\ln(1-\alpha )\right]}^{(n-1)/n}$$

(7) ln[−ln(1−α)]=lnk+nlnt

where *α* refers to the degree of conversion; *K* denotes the kinetic constant; *k* equals to *K^n^*; and *n* is the kinetic parameter; *t* represents the time. Equation (7) is an equation of a straight line with slope *n* in the coordinates ln (−ln (1 − *α*)) vs. ln *t*. If the value of *n* is higher than 1, the absorption reaction is controlled by the formation and growth of product crystals. When *n* equals to 0.5 approximately, the absorption reaction is controlled by the diffusion of ions [31].

**Figure 7:**
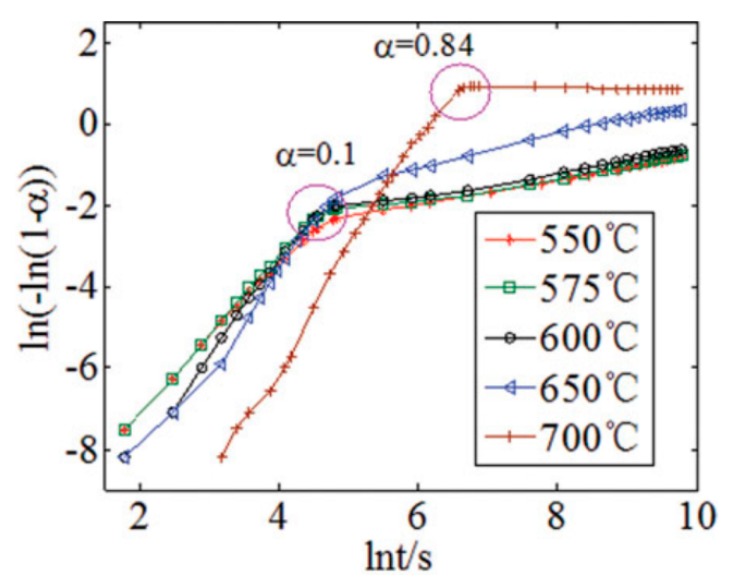
Fit of kinetic experimental data by the Avrami–Erofeev model [27].

As illustrated in Figure 7, the curves of Avrami–Erofeev model look similar to TGA curves obtained from 550 to 700 °C, and the rapid chemical reaction-controlled stage and the slow diffusion-controlled stage can be easily distinguished at every temperature. Additionally, Zhang et al. [27] reported that the Avrami–Erofeev model suited the regeneration process of Li_4_SiO_4_ material, and the entire regeneration process was controlled by the rate of the formation and growth of product crystals, which was also confirmed by Xiang et al. [32]. Thus, the Avrami–Erofeev model is more suitable for CO_2_ absorption by Li_4_SiO_4_.

## 3. Synthesis of Li_4_SiO_4_ Materials with Superior Cyclic Absorption/Regeneration Performance

It is clear that Li_4_SiO_4_ material synthesized by the solid-state reaction method from SiO_2_ and Li_2_CO_3_ achieves low CO_2_ absorption capacity, due to the low porosity of Li_4_SiO_4_ generated at high temperatures during the preparation. Thus, the CO_2_ absorption capacity of Li_4_SiO_4_ decreases rapidly with the number of cycles, which is the main disadvantage of Li_4_SiO_4_ material in industrial applications. The various methods have been reported to enhance the cyclic performance of Li_4_SiO_4_ material prepared by solid-state reaction method, such as hydration [33] and ball milling [34,35]. The strategies to enhance the cyclic performance of Li_4_SiO_4_ can be categorized as follows: (i) reducing the diffusion resistance by adding solid solutions or molten salts; (ii) using alternative precursors; and (iii) using a more appropriate synthesis method.

### 3.1. Modification for Li_4_SiO_4_ Prepared by Solid-State Reaction

As discussed above, high temperature during the preparation by the solid-state reaction method of Li_4_SiO_4_ leads to sintering, and the core of Li_4_SiO_4_ usually cannot react with CO_2_. Thus, Yin et al. [33] proposed a hydration process to improve the pore structure of Li_4_SiO_4_ material. First, Li_4_SiO_4_ material was prepared by the solid-state reaction method, and then distilled water was added to the samples and stirred at 80 °C for 4 h. They reported that dense particles formed during the solid-state reaction preparation could be split into fine particles, so the porous structure and high cyclic CO_2_ absorption capacity of Li_4_SiO_4_ was obtained by hydration process.

Romero-Ibarra et al. [34] used the ball milling process to modify the surface properties of Li_4_SiO_4_ material, and they found that the ball milling process decreased the particle size and improved the surface area of Li_4_SiO_4_ material. Kanki et al. [35] reported that the ball milling process could promote CO_2_ absorption of Li_4_SiO_4_ material at lower temperatures, and longer ball milling duration led to higher CO_2_ absorption capacity. Additionally, the doping of K_2_CO_3_ in Li_4_SiO_4_ material improved its CO_2_ absorption capacity under short ball milling duration.

### 3.2. Doping of Solid Solutions or Molten Salts

#### 3.2.1. Solid Solutions

The CO_2_ absorption rate of Li_4_SiO_4_ material is mainly controlled by the diffusion of ions and CO_2_. Zhao et al. [36] reported that solid solution usually formed with the doping of Al_2_O_3_ during solid-state preparation, thus increased oxygen vacancies could promote the diffusion in the product layer. Ortiz-Landeros et al. [37] reported that Al_2_O_3_ addition and ball milling could extend the range of CO_2_ absorption temperature. In addition, Ortiz-Landeros et al. [38] compared Li_4+x_(Si_1−x_Al_x_)O_4_ with Li_4−x_(Si_1−x_V_x_)O_4_ as the solid solutions, and the results showed that diffusion resistance of CO_2_ and ions in Li_4+x_(Si_1−x_Al_x_)O_4_ was diminished, while the presence of V was adverse to the diffusion through the product layer.

#### 3.2.2. Molten Salts

The doping of alkali metals, such as Na and K, could produce a layer of molten salts with low eutectic temperature, which reduced diffusion resistance effectively, thus the limiting step of Li_4_SiO_4_ material for CO_2_ absorption could be resolved. The CO_2_ absorption performance of various alkali metal-doped Li_4_SiO_4_ materials is summarized in Table 2 [39,40,41,42,43,44,45,46].

**Figure 8:**
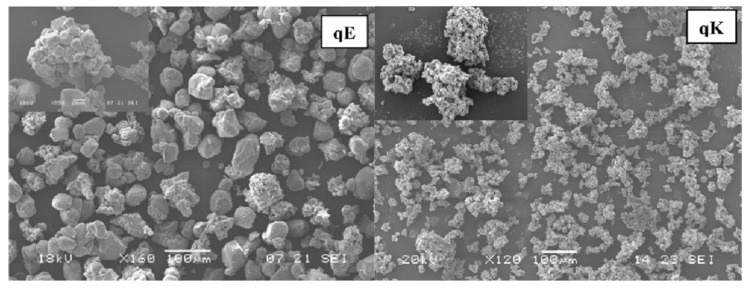
Scanning electron microscopy (SEM) images of Li_4_SiO_4_ samples obtained from different doping methods: eutectic doping (qE); simple mechanical addition (qK) [39].

As presented in Table 2, Na and K were the most commonly reported alkali metals to enhance the CO_2_ absorption performance of Li_4_SiO_4_ material. In order to determine the most appropriate doping method for K_2_CO_3_, Seggiani et al. [39] compared eutectic doping and simple mechanical addition of K_2_CO_3_, and they found that Li_4_SiO_4_ particles obtained from mechanical addition were smaller, as shown in Figure 8, so the mechanical doping method may be more appropriate for the doping of K_2_CO_3_. Olivares-Marín [47] et al. synthesized K_2_CO_3_-doped Li_4_SiO_4_ material with fly ash as the silicon precursor, and they reported that the CO_2_ absorption capacity of the prepared Li_4_SiO_4_ material increased with the increase of the dopant amount. It is also worth noting that Zhang et al. [42] reported that the K_2_CO_3_ doped Li_4_SiO_4_ material cooperated well with the Ni/γ-Al_2_O_3_ catalyst in the sorption-enhanced steam methane reforming (SE-SMR) system, and high-purity hydrogen (>95%) could be obtained at lower temperatures ranging from 500 to 550 °C, and the presence of steam in the regeneration atmosphere could improve the reaction rate obviously. Mejía-Trejo et al. [48] prepared Na-doped Li_4_SiO_4_ material by doping Na_2_CO_3_ into the starting materials of TEOS and Li_2_CO_3_ through the co-precipitation route, and they noted that the addition of Na_2_CO_3_ increased the activity and reduced the equilibrium temperature of Li_4_SiO_4_ material for CO_2_ absorption, and Li_3.85_Na_0.15_SiO_4_ had the highest CO_2_ absorption capacity among various Na-doped Li_4_SiO_4_ materials. Seggiani et al. [40] noted that dopants like K_2_CO_3_ and Na_2_CO_3_ could form eutectic mixtures with Li_2_CO_3_, which melted at high temperatures (>500 °C), so the diffusion of ions and CO_2_ was enhanced in the diffusion-controlled stage. Yang et al. [43] reported that orderly crystalline arrangement of Li_4_SiO_4_ was broken by doped K_2_CO_3_ and Na_2_CO_3_ for their different crystal sizes, thus more pores and larger specific surface area were generated.

#### 3.2.3. Other Dopants

**Figure 9:**
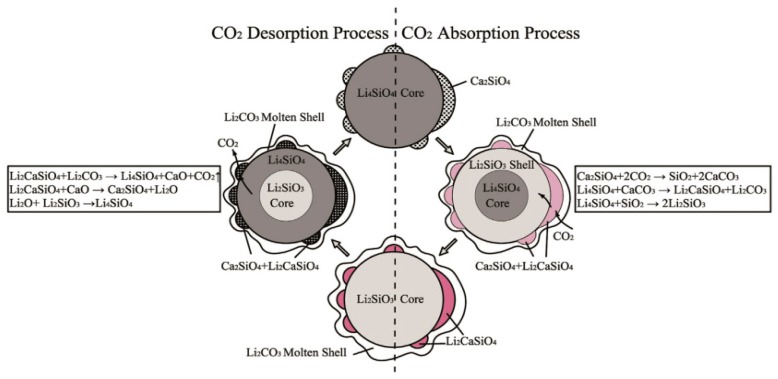
CO_2_ absorption and regeneration mechanism of Ca-doped Li_4_SiO_4_ [51].

Wang et al. [49] prepared K-, Mg-, Cr-, and Ce-doped Li_4_SiO_4_ and found that Ce was the most difficultly doped into the Li_4_SiO_4_ crystal lattice among the four elements. However, Ce was the most effective to inhibit the aggregation of Li_4_SiO_4_ grains, so Ce-doped Li_4_SiO_4_ achieved the highest CO_2_ absorption performance. Subha et al. [50] studied the CO_2_ absorption by Li_4_SiO_4_ material doped with Y_2_O_3_, Gd_2_O_3_ or LaPO_4_, and found that both Y_2_O_3_ and Gd_2_O_3_ improved the CO_2_ absorption capacity of Li_4_SiO_4_, and Y_2_O_3_-doped Li_4_SiO_4_ retained the highest CO_2_ absorption capacity due to the segregation of second phase created by the doped unreacted Y_2_O_3_. Chen et al. [51] reported that Ca-doped Li_4_SiO_4_ material achieved high CO_2_ absorption capacity and they proposed a modified double-shell mechanism to describe the CO_2_ absorption and regeneration mechanism of Ca-doped Li_4_SiO_4_ as shown in Figure 9. The transformation of Ca_2_SiO_4_ to Li_2_CaSiO_4_ during CO_2_ absorption process was beneficial of transferring CO_2_ from Li_4_SiO_4_ surface to the core, which reduced the diffusion resistance and improved CO_2_ absorption, and regeneration was also correspondingly enhanced.

Additionally, doping of organic matter can also enhance the CO_2_ absorption property of Li_4_SiO_4_ material due to the formation of the porous structure. Wang et al. [30,52] prepared carbon-coated Li_4_SiO_4_ material by the sol-gel method, and gluconic acid and citric acid were used as the complexing agents, respectively. During the carbonization stage, gluconic acid and citric acid decomposed, and a mesoporous carbon coating covered the surface of Li_4_SiO_4_ material, which suppressed the growth of Li_4_SiO_4_ grains. As a result, the cyclic CO_2_ absorption capacities and rates of carbon-coated Li_4_SiO_4_ materials were higher than that of uncoated Li_4_SiO_4_ during multiple absorption/regeneration cycles. Furthermore, CMK-3, as a kind of porous carbon material [53], was also introduced into the Li_4_SiO_4_ material. Jeoung et al. [54] prepared CMK-modified Li_4_SiO_4_, while the cyclic absorption capacity of CMK-modified Li_4_SiO_4_ decreased obviously with the number of cycles.

It has been reviewed in this part that doping of metal elements, such as K, Na, Ca, Ce, Y, Al, or organic matters, can enhance the CO_2_ absorption capacities of Li_4_SiO_4_ material. The limitation in the diffusion-controlled stage for Li_4_SiO_4_ is reduced greatly with the doping of solid solution or molten salts, and the porous structure of Li_4_SiO_4_ by doping of organic matters is obtained. The additive amounts are minor, but the CO_2_ absorption performance of Li_4_SiO_4_ can be greatly enhanced.

### 3.3. Selection of Alternative Precursors for Preparation of Li_4_SiO_4_

Li_4_SiO_4_ material is usually prepared from Li_2_CO_3_ and SiO_2_, which are not able to create a favorable surface characteristic for CO_2_ absorption. Recent studies have shown that Li_4_SiO_4_ materials prepared from alternative precursors, especially organic precursors, rather than Li_2_CO_3_ and SiO_2_ achieve high CO_2_ absorption capacities and cyclic stability. In this section, the effects of precursors on CO_2_ absorption capacity of Li_4_SiO_4_ material are summarized.

#### 3.3.1. Lithium Precursors

Kim et al. [29] synthesized Li_4_SiO_4_ material from LiOH and fumed silicate by the solid-state reaction method. They reported that the synthesis temperature could be reduced to 600 °C due to the use of LiOH, and the obtained Li_4_SiO_4_ showed higher CO_2_ absorption capacity compared with those synthesized at 700 °C and 800 °C, which achieved 298 mg/g after 10 cycles. Wang et al. [55] synthesized Li_4_SiO_4_ with LiOH by the sol-gel technique and they found that LiOH-synthesized Li_4_SiO_4_ particles were primarily composed of porous grains, and the average grain size of Li_4_SiO_4_ prepared by the sol-gel method was much smaller than that synthesized by the solid-state reaction method.

Weng et al. [56] synthesized Li_4_SiO_4_ from LiNO_3_ as lithium precursor and tetraethyl orthosilicate (TEOS) as a silicon precursor by the sol-gel method. The CO_2_ absorption capacity of the obtained Li_4_SiO_4_ material increased with increasing temperature from 400 to 500 °C in 2% CO_2_. Bretado et al. [14] reported that the solid-state reaction method was more appropriate than the sol-gel method for the preparation of Li_4_SiO_4_ material when LiNO_3_ was used as the lithium precursor. However, Subha et al. [25] reported that the sol-gel method was superior to the solid-state reaction method for Li_4_SiO_4_ material prepared from LiNO_3_ and colloidal silica. This indicates that the most appropriate synthesis method depends on the lithium and silicon precursors simultaneously.

**Figure 10:**
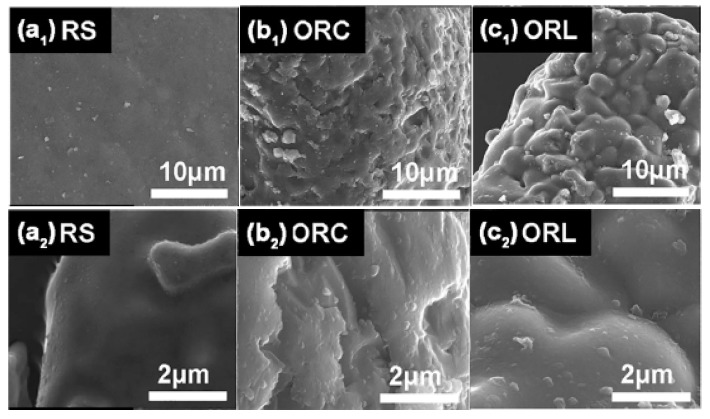
SEM images of three kinds of Li_4_SiO_4_ materials prepared with Li_2_CO_3_ (RS), lithium acetate (ORC) and lithium lactate (ORL) [19].

Compared with inorganic lithium precursors, organic lithium-containing materials seems more appropriate as the lithium precursor for the preparation of Li_4_SiO_4_ material. Yang et al. [19] used lithium acetate and lithium lactate to prepare novel Li_4_SiO_4_ materials by the impregnated suspension method. As shown in Figure 10, the two novel Li_4_SiO_4_ materials showed a bulgier morphology and more porous structure, compared with Li_4_SiO_4_ synthesized by the solid-state reaction method. Absorption capacities of Li_4_SiO_4_ material prepared from lithium acetate or lithium lactate as the lithium precursors were almost six times higher than that of a conventional Li_4_SiO_4_ material. Additionally, the CO_2_ absorption capacities and conversions of Li_4_SiO_4_ material prepared from lithium acetate or lithium lactate showed an incremental tendency over 40 cycles, and the conversion of Li_4_SiO_4_ prepared from lithium acetate was approximately 70% even in the last cycle, which was calculated according to Equation (8):

(8) $$X_{N}=\frac{C_{N}}{m_{0}}$$

where *X_N_* is the conversion of Li_4_SiO_4_ during the *N*th cycle, %; and *m*_0_ is the theoretical CO_2_ absorption capacity of Li_4_SiO_4_ material, which is 367 mg/g. Lee et al. [57] used Li and a Si-containing metal-organic framework (MOF) as the silicon precursor, and the prepared Li_4_SiO_4_ material was able to convert into Li_4_SiO_4_ thermally. The as-prepared material had a coral-like morphology, so the contact area between CO_2_ and Li_4_SiO_4_ material was enhanced, and the Li_4_SiO_4_ material showed higher CO_2_ absorption capacity than that prepared by the conventional solid-state reaction method.

In this section, CO_2_ absorption performance of Li_4_SiO_4_ material synthesized from various lithium sources was reviewed. It is known to all that the price of Li-containing materials is quite high now, including Li_2_CO_3_, LiOH, LiNO_3_ or organic lithium precursors mentioned above, so it is necessary to find other alternative Li-containing materials, especially wastes, with lower prices as the lithium precursor for the preparation of Li_4_SiO_4_ material.

#### 3.3.2. Silicon Precursors

SiO_2_ is an essential raw material for the synthesis of Li_4_SiO_4_. In addition to pure SiO_2_, there are many SiO_2_-rich industrial wastes which have attracted researchers’ interests, such as rice husk ash (RHA) and fly ash (FA). In this section, the effects of alternative silicon precursors on CO_2_ absorption performance of Li_4_SiO_4_ material are critically reviewed.

Wang et al. [20] selected two kinds of RHA samples as the silicon precursors for the preparation of Li_4_SiO_4_ material, which contained the SiO_2_ contents of 94.71 and 98.84 wt.%, respectively. HCl aqueous solution was used to pretreat the two RHA samples, then Li_4_SiO_4_ materials were synthesized by the solid-state reaction method with Li_2_CO_3_. The employment of RHA produced a smaller particle size, larger pore volume, and surface area compared with pure Li_4_SiO_4_ material. They reported a weight gain of nearly 135 % over 15 cycles, which was much higher than that of pure Li_4_SiO_4_ material. Furthermore, Wang et al. [58] pretreated rice husk samples at 600 and 1000 °C, respectively, and cyclic performances of the two RHA-synthesized Li_4_SiO_4_ materials pretreated at 1000 °C achieved better CO_2_ absorption performance, which was similar to that of the RHA-derived Li_4_SiO_4_ material mentioned above. To study the effects of RHA as the silicon precursor on the CO_2_ absorption properties of Li_4_SiO_4_ material, Wang et al. [59] selected RHA and two kinds of nanosilica (Aerosil and quartz) to prepare Li_4_SiO_4_ materials by solid-state reaction method, and SEM images and BET analysis indicated that RHA-synthesized Li_4_SiO_4_ material possessed higher surface area and larger pore volume. Furthermore, the weight gain of RHA-synthesized Li_4_SiO_4_ material was higher and faster than that of the two nanosilica-synthesized Li_4_SiO_4_ materials, and its cyclic CO_2_ absorption capacity reached nearly 30 wt.% over 15 cycles. The authors ascribed this phenomenon to the almost unchanged surface morphology of Li_4_SiO_4_ material prepared from RHA over multiple absorption/regeneration cycles. Qiao et al. [60] also noted that RHA-derived Li_4_SiO_4_ material could enhance the yield of H_2_ and reduce the energy consumption in the process of sorption-enhanced steam ethanol reforming.

Fly ash (FA) is a kind of hazardous mineral residue released from coal-fired power plants, and it accounts for approximately 88% in the total coal ash content, which contains a high silicon content, thus it has been used to fabricate useful materials [61,62]. Therefore, Li_4_SiO_4_ materials can also be prepared from FA as a silicon precursor. Olivares-Marín et al. [47] fabricated Li_4_SiO_4_ material from Li_2_CO_3_ and three kinds of FA, and the samples were doped with several amounts ranging from 5 to 40 mol% of K_2_CO_3_. The cyclic CO_2_ absorption capacity of one of the doped FA-Li_4_SiO_4_ was approximately 100 mg/g over 10 cycles, which was far below the theoretical absorption capacity of Li_4_SiO_4_ material synthesized from pure SiO_2_, but it was relatively stable over multiple cycles. Sanna et al. [63] synthesized Na/Li-FA Li_4_SiO_4_ material with different molar ratios of Li_2_CO_3_, FA, and Na_2_CO_3_, and the material was doped with K_2_CO_3_. They reported that the CO_2_ absorption capacity of the obtained Li_4_SiO_4_ material was approximately 50 mg/g in low CO_2_ concentration in the presence of water vapor, and water vapor had no effect on the cyclic CO_2_ absorption capacity.

Shan et al. [64] selected diatomite as silicon precursor, containing the SiO_2_ content of approximately 75% [65], and zeolite was also chosen as precursor for comparison. Li_4_SiO_4_ was synthesized by the solid-state reaction method. Li_4_SiO_4_ synthesized from diatomite showed higher CO_2_ absorption capacity. Li_4_SiO_4_ material synthesized from diatomite achieved better CO_2_ absorption performance than that synthesized from pure SiO_2_ because of the higher specific surface area of the former [66]. In order to determine the optimum molar ratio of Li_2_CO_3_ to SiO_2_, Shan et al. [65] prepared a series of Li_4_SiO_4_ containing the molar ratios of Li_2_CO_3_ to SiO_2_ ranging from 2.0 to 2.8 and their CO_2_ absorption capacities carbonated under 50 vol.% CO_2_ at 620 °C for 30 min were shown in Table 3.

As presented in Table 3, when molar ratio of Li_2_CO_3_ to SiO_2_ was 2.6:1, CO_2_ absorption capacity reached 30.32 wt.% (82.62% of the theoretical value). The CO_2_ absorption capacity of Li_4_SiO_4_ material with this molar ratio decreased from 34.14 to 27.70 wt.% over 16 cycles. However, Shan et al. [67] pointed out that high temperature (900 °C) during the solid-state reaction preparation process resulted in the sintering of Li_4_SiO_4_ easily, so they selected the impregnation precipitation method to prepare Li_4_SiO_4_ materials, which was operated at lower temperature. Diatomite, LiNO_3_, and NH_3_·H_2_O were selected as the starting materials with the Li:Si molar ratio of 5.2:1, and the reactions involved are shown in Equations (9) and (10). When carbonated in 50 vol.% CO_2_ and regenerated in pure N_2_ at 700 °C, both for 30 min, cyclic CO_2_ absorption capacity of Li_4_SiO_4_ synthesized by the impregnation precipitation method was quite stable, which decreased from 34.14 to 33.09 wt.% as the cycle number increases from 1 to 15.

(9) $${\mathrm{LiNO}}_{3}+{\mathrm{NH}}_{3}\cdot H_{2}O\to \mathrm{LiOH}+{\mathrm{NH}}_{4}{\mathrm{NO}}_{3}$$

(10) $$4\mathrm{LiOH}+{\mathrm{SiO}}_{2}\to {\mathrm{Li}}_{4}{\mathrm{SiO}}_{4}+2H_{2}O$$

Halloysite is also a SiO_2_-containing material with a SiO_2_ content of about 50 wt.% [68]. Niu et al. [69] synthesized Li_4_SiO_4_ from treated halloysite nanotubes (HNTs) with HCl aqueous solution and Li_2_CO_3_ by the solid-state reaction method at 800 °C. The content of Al_2_O_3_ of HNTs is 43.859%, and the presence of Al^3+^ was beneficial to the enlargement of Li_4_SiO_4_ crystalline structure, which is beneficial for its CO_2_ absorption performance [37]. The CO_2_ absorption capacity of halloysite-synthesized Li_4_SiO_4_ material was approximately 30 wt.% over 10 cycles, which was higher than that of SiO_2_-synthesized Li_4_SiO_4_ material.

In this section, CO_2_ absorption performances of Li_4_SiO_4_ materials synthesized from various silicon precursors were reviewed. Li_4_SiO_4_ synthesized from RHA, diatomite and halloysite exhibited high CO_2_ absorption capacity, while fly ash was not a good lithium precursor. Some elements in these silicon precursors other than pure SiO_2_ are possibly beneficial for the CO_2_ absorption properties of Li_4_SiO_4_ materials, which will be discussed in the following sections. There are a large number of Si-containing materials, especially industrial wastes like steel slag, so the following research will focus on these materials. The studies on alternative silicon precursors for the preparation of Li_4_SiO_4_ materials have obtained great progress, while the major problem that limits the practical application of Li_4_SiO_4_ is the high price of Li-containing materials, and the cost of Li_4_SiO_4_ will not be reduced by much even if SiO_2_ is free of charge, so the future research should focus on alternative lithium precursors.

#### 3.3.3. Synthesis Methods

Most of the Li_4_SiO_4_ materials were synthesized by the traditional solid-state reaction method at a relatively high temperature (900 °C). The solid-state reaction method has been widely used because of its simplicity, while Bretado et al. [14] reported that high temperature during the solid-state reaction process resulted in contamination and volatilization. In addition, the microstructure and composition of Li_4_SiO_4_ materials were difficult to control and agglomeration and sintering of the materials also occurred in the preparation process [24,29]. Thus, Bretado et al. [14] selected the impregnated suspension method to prepare Li_4_SiO_4_ material and they found that the conversion of the obtained Li_4_SiO_4_ material (98.4%) was higher than that prepared by the solid-state reaction method (94.9%).

Subha et al. [25] reported that the platelet-shaped Li_4_SiO_4_ material synthesized from LiNO_3_ and colloidal silica by a sol-gel method achieved an absorption capacity of 350 mg/g. Additionally, the platelet-shaped Li_4_SiO_4_ material was coated with a porous carbon mesh, and the cyclic absorption/regeneration performance of the platelet-shaped Li_4_SiO_4_ material retained approximately 120 mg/g over eight cycles. The CO_2_ absorption rates of the coated Li_4_SiO_4_ materials were faster than those of the uncoated ones. Additionally, the sol-gel method was superior to the solid-state reaction method when LiOH was selected as the lithium precursor [29,55]. However, the impregnation precipitation method was superior to the solid-state reaction method when diatomite was selected as the silicon precursor [67]. Venagas et al. [70] reported that Li_4_SiO_4_ materials synthesized by the sol-gel method was not completely pure, probably because the use of a microwave oven resulted in the sublimation of Li_4_SiO_4_.

## 4. Effects of Particle Properties on CO_2_ Absorption Performance of Li_4_SiO_4_ Material

The newly synthesized Li_4_SiO_4_ powder is too fine, and elutriation might occur in the reactor, especially in fluidized bed reactors, in industrial applications. In addition, powdery Li_4_SiO_4_ materials cannot create effective fluidization, while most of the studies on CO_2_ absorption by Li_4_SiO_4_ material were conducted on fixed bed reactors or TGA. Thus, pelletization may be an effective method for the practical application of Li_4_SiO_4_ materials. The effects of the particle properties on CO_2_ absorption performance of Li_4_SiO_4_ material were critically reviewed in this section.

**Figure 11:**
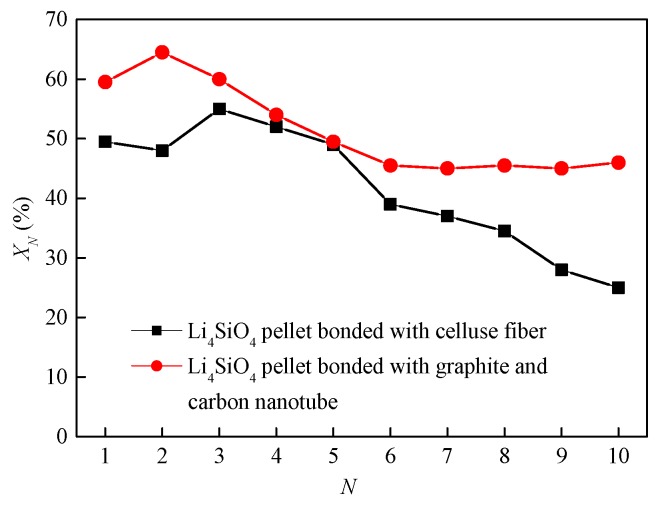
Cyclic CO_2_ absorption by Li_4_SiO_4_ materials with different binders [74,75].

Pacciani et al. [71] studied the CO_2_ absorption by the pelletized Li_4_SiO_4_ materials, which were doped with less than 10 vol.% K_2_CO_3_ and Li_2_TiO_3_ as a binder. The CO_2_ absorption capacity of the pelletized Li_4_SiO_4_ material was 23 wt.% carbonated in 10 vol.% CO_2_. However, Kato et al. [72] reported that the pelletized Li_4_SiO_4_ materials were more prone to lose their cyclic stability due to the sintering which was caused by the short length of material particles. Essaki et al. [73] prepared cylinder-type K_2_CO_3_ doped Li_4_SiO_4_ materials with the diameter of 3 mm and length of 6 mm, while the CO_2_ absorption capacity of a Li_4_SiO_4_ pellet was not so high as that of Li_4_SiO_4_ powder. Puccini et al. [74] synthesized K_2_CO_3_-doped Li_4_SiO_4_ by the solid-state reaction method, and they selected cellulose fiber as the binder. The Li_4_SiO_4_ material pellets with a diameter of 6 mm and lengths of 1.5, 2.5, and 3.5 mm were prepared, but the prepared Li_4_SiO_4_-based pellets did not show superior cyclic performance and the conversion of the Li_4_SiO_4_ pellets decreased to below 28% after 10 cycles. Furthermore, Puccini et al. [75] selected layered graphite and carbon nanotubes as the binders, and thermogravimetric analysis showed that layered graphite was a more suitable binder than carbon nanotubes. It is noteworthy that the cyclic performance of Li_4_SiO_4_ pellets with a binder of layered graphite was more superior than that of the pellets mentioned in [74], as shown in Figure 11.

Pelletization is an essential procedure for the practical application of Li_4_SiO_4_, but few researchers studied the CO_2_ absorption performance of the pelletized Li_4_SiO_4_ materials in fluidized bed reactors. Additionally, mechanical intensity and wearing characteristics of pelletized Li_4_SiO_4_ materials have seldom been reported.

## 5. Effects of Reaction Conditions on CO_2_ Absorption Performance of Li_4_SiO_4_ Material

Realistic reaction conditions for CO_2_ absorption by Li_4_SiO_4_ material is very complicated, which involves the absorption atmosphere, absorption temperature, regeneration (desorption) temperature, and operating pressure, etc. Hence, the effects of reaction conditions on the CO_2_ absorption performance of Li_4_SiO_4_ materials are reviewed in this section.

### 5.1. Reaction Atmosphere

#### 5.1.1. CO_2_ Concentration

The practical CO_2_ concentration in the flue gas from fossil fuel-fired power plant is about 15 vol.% [76], but pure CO_2_ is usually selected as the absorption atmosphere of Li_4_SiO_4_, and the CO_2_ absorption performance under the practical lower CO_2_ concentration has been overlooked. In fact, CO_2_ concentration in sorption-enhanced hydrogen production process is also usually low. Therefore, it is necessary to investigate the CO_2_ absorption performance of Li_4_SiO_4_ material in low CO_2_ concentrations.

**Figure 12:**
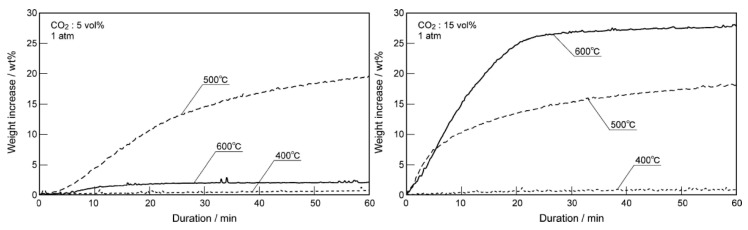
Weight increase of Li_4_SiO_4_ at different temperatures in different CO_2_ concentrations [77].

Pacciani et al. [71] reported that the CO_2_ absorption rate of Li_4_SiO_4_ material rose apparently when CO_2_ concentration in absorption atmosphere increased from 2.5 to 24.5 vol.%. Essaki et al. [77] prepared the pelletized Li_4_SiO_4_ materials with an average particle size of 5 mm and K_2_CO_3_ and Li_2_ZrO_3_ were doped into the materials to promote the absorption reaction and prevent reduction of absorption capacity, respectively. The absorption property of Li_4_SiO_4_ pellets was investigated in 5 vol.% CO_2_ at first, and they found that 500 °C was the most appropriate temperature in the range of 400–600 °C for the CO_2_ absorption by Li_4_SiO_4_. However, when the absorption tests were carried out in 10 or 15 vol.% CO_2_, it was found that the CO_2_ absorption capacity rose as the temperature increased from 400 to 600 °C. Essaki et al. [77] ascribed this phenomenon to the influence of reaction equilibrium, as shown in Figure 12. The equilibrium temperature of CO_2_ absorption and regeneration showed an increasing trend with increasing CO_2_ concentration, and the weight increase was used to evaluate the CO_2_ absorption performance of Li_4_SiO_4_ material, which can be calculated according to Equation (11):

(11) $$I_{N}=W_{N}-100\%$$

where *I_N_* is the weight increase of Li_4_SiO_4_ material during the *N*th cycle, wt.%; *W_N_* is the weight gain, wt.%; *N* is the number of cycles. It was also noteworthy that the CO_2_ absorption process of Li_4_SiO_4_ was limited in low CO_2_ concentration (5 vol.%), while it was controlled by the diffusion of Li^+^ and O^2−^ in high CO_2_ concentration (15 vol.%).

Researchers found that the limits of low CO_2_ concentration could be counteracted by the addition of dopants. Puccini et al. [78] found Li_4_SiO_4_ material doped with 30 wt.% K_2_CO_3_ maintained a stable CO_2_ absorption capacity (approximately 160 mg/g) after 25 cycles in 4 vol.% CO_2_ at 580 °C. It is worth noting that Seggiani et al. [39] reported that CO_2_ absorption capacity of K_2_CO_3_-doped Li_4_SiO_4_ material was superior than 20 wt.% over four cycles in 4 vol.% CO_2_. Furthermore, Seggiani et al. [40] also reported that the CO_2_ absorption capacity of Na_2_CO_3_-doped Li_4_SiO_4_ material in 4 vol.% CO_2_ was 7 wt.%, and it was quite stable over 25 cycles. Adding some dopants can improve the CO_2_ absorption capacity of Li_4_SiO_4_ material, but the improvement in lower CO_2_ concentration is still relatively lower compared with that in higher CO_2_ concentration. The CO_2_ absorption performance of Li_4_SiO_4_ material in high CO_2_ concentration has been well studied by researchers. Thus, Li_4_SiO_4_ materials with high absorption capacity, fast absorption rate, and good cyclic stability in low CO_2_ concentrations should be investigated for industrial application.

#### 5.1.2. Presence of Steam

Apart from CO_2_, steam also exists in realistic CO_2_ absorption conditions, and the content of steam during the typical sorption-enhance hydrogen production process is more than 30%. Ochoa-Fernández et al. [79] reported that steam could promote the mobility of alkaline ions, indicating that the limiting resistance of the CO_2_ absorption reaction could be reduced. Thus, the presence of steam in the absorption atmosphere also has non-negligible effect on the CO_2_ absorption capacity of Li_4_SiO_4_ material.

Ochoa-Fernández et al. [80] reported that the presence of 10 vol.% steam in the absorption atmosphere could raise the CO_2_ capacity from 9.5 to 29 wt.%. Additionally, they also found that the presence of steam accelerated the regeneration reaction: the regeneration process became faster and more thorough with the presence of steam, and cyclic CO_2_ absorption performance of Li_4_SiO_4_ material degraded slightly after eight cycles with the presence of steam, almost the same as the experimental data obtained in dry atmosphere. Quinn et al. [81] used pelletized Li_4_SiO_4_ materials for CO_2_ absorption in 14.7% CO_2_, 2.6% steam in N_2_ at 550 °C, and they found that the CO_2_ absorption capacity after 10 min was almost three times higher than that in dry atmosphere. Furthermore, Sanna et al. [63] synthesized Li_4_SiO_4_ material from FA as SiO_2_ precursor, and the CO_2_ absorption capacity was enhanced by steam. Puccini et al. [82] also noted that the CO_2_ absorption rate was accelerated correspondingly with increasing steam concentration from 10 to 30 vol.%.

As mentioned above, the presence of steam contributes to the CO_2_ absorption by Li_4_SiO_4_ material, because the addition of steam maybe enhances the mobility of Li^+^ and O^2−^ [27,83], thus the resistance of diffusion is reduced, so the CO_2_ absorption capacity of Li_4_SiO_4_ is enhanced by steam.

#### 5.1.3. Gas Contaminants

NO_x_ and SO_2_ are common gas contaminants which have done great harm to the environment and people’s health. Thus, the effects of NO_x_ and SO_2_ in the flue gas on the CO_2_ absorption by Li_4_SiO_4_ material should be considered. The effects of NO_x_ and SO_2_ on the CO_2_ absorption performance of Li_4_SiO_4_ material could be great despite of their minor contents [82].

Puccini et al. [82] performed CO_2_ absorption tests in an atmosphere of 4 vol.% CO_2_ and various concentrations of NO, and the results showed that NO in the absorption atmosphere does not show a harmful effect on the CO_2_ absorption capacity of Li_4_SiO_4_ material. Furthermore, when the concentration of SO_2_ in the absorption atmosphere increased from 0 to 2000 ppm, the weight change of Li_4_SiO_4_ material increased with the increase of SO_2_ concentration, but the regeneration performance of Li_4_SiO_4_ material in the presence of SO_2_ was worse compared with that in the absence of SO_2_. Additionally, the cyclic CO_2_ absorption performance of Li_4_SiO_4_ material was negatively influenced in the presence of SO_2_ under absorption and regeneration atmospheres [71]. The authors ascribed this phenomenon to the nonreversible reaction between SO_2_ and Li_4_SiO_4_, as shown in Equations (12) and (13):

(12) $${\mathrm{Li}}_{4}{\mathrm{SiO}}_{4}{+\mathrm{SO}}_{2}\to {\mathrm{Li}}_{2}{\mathrm{SO}}_{3}{+\mathrm{Li}}_{2}{\mathrm{SiO}}_{3}$$

(13) $${\mathrm{Li}}_{2}{\mathrm{SiO}}_{3}{+\mathrm{SO}}_{2}\to {\mathrm{Li}}_{2}{\mathrm{SO}}_{4}+\mathrm{SiO}$$

The formation of Li_2_SO_3_ and Li_2_SO_4_ prevented the regeneration of the materials, indicating that the presence of SO_2_ in the absorption atmosphere has an adverse effect on the absorption performance of Li_4_SiO_4_ material [82]. In general, NO had no negative impact on the CO_2_ absorption property of Li_4_SiO_4_ materials, while SO_2_ had an adverse effect due to the formation of the irreversible Li_2_SO_3_ and Li_2_SO_4_, so SO_2_ must be scrubbed prior to the trapping of CO_2_. However, the exact joint role and acting mechanism of NO and SO_2_ in the process of CO_2_ capture are still unknown, and the effects of other contaminants, like HCl or H_2_S, on the CO_2_ absorption by Li_4_SiO_4_ material are not clear, thus further research is necessary.

### 5.2. Reaction Temperature

As shown in Figure 4, the equilibrium temperatures of absorption and regeneration increase as the CO_2_ partial pressure rises monotonously. In other words, each equilibrium temperature corresponds with a partial pressure of CO_2_ in the absorption atmosphere. Essaki et al. [77] reported that when the absorption temperature of Li_4_SiO_4_ pellets provided by Toshiba varied from 400 to 600 °C in 5 vol.% CO_2_, and results showed that weight increase at 500 °C was 20 wt.%, which was much higher than those at 400 °C and 600 °C. Additionally, Quinn et al. [81] reported that 625 °C was the most appropriate temperature for the absorption of Toshiba-provided Li_4_SiO_4_ pellets in a pure CO_2_ atmosphere. This confirmed the conclusion that the equilibrium temperature of the reaction between Li_4_SiO_4_ and CO_2_ rises with increasing CO_2_ partial pressure.

Different kinds of Li_4_SiO_4_ materials accommodate diverse appropriate absorption temperatures. Qiao et al. [60] synthesized Li_4_SiO_4_ material from RHA and Li_2_CO_3_, and they found that the most suitable temperature for absorption was 650 °C in a pure CO_2_, while Puccini et al. [78] reported that 580 °C was the optimum temperature for K-doped Li_4_SiO_4_ materials, and Wang et al. [30] pointed out that 575 °C was the most appropriate for the CO_2_ absorption by Li_4_SiO_4_.

Temperature during the regeneration process also had a deep effect on CO_2_ absorption performance on Li_4_SiO_4_ material. Ochoa-Fernández et al. [80] reported that the ratio and degree of regeneration increased when the regeneration temperature rose from 525 to 575 °C. This indicates that a higher regeneration temperature is possibly advantageous for the regeneration of Li_4_SiO_4_ material, while too high a regeneration temperature intensifies the sintering of the material, which is extremely harmful.

## 6. Application of Li_4_SiO_4_ Material in Sorption-Enhanced Hydrogen Production

Sorption-enhanced hydrogen production is one of the most important applications of Li_4_SiO_4_ material as a CO_2_ acceptor, which mainly consists of sorption-enhanced steam methane reforming (SE-SMR) and sorption-enhance steam ethanol reforming (SE-SER). Overall reactions of SE-SMR and SE-SER are shown in Equations (14) and (15), respectively:

(14) $${\mathrm{CH}}_{4}{+2H}_{2}O\to {\mathrm{CO}}_{2}{+4H}_{2}$$

(15) $$C_{2}H_{5}{\mathrm{OH}+3H}_{2}O\to 2{\mathrm{CO}}_{2}{+6H}_{2}$$

In the SE-SMR and SE-SER processes, in situ CO_2_ removal of Li_4_SiO_4_ material as the CO_2_ acceptor shifts the reaction equilibrium to hydrogen production, and exothermal absorption of CO_2_ by the Li_4_SiO_4_ material provides heat for reforming, thus high hydrogen yield can be achieved.

Rusten et al. [84] conducted SE-SMR with CO_2_ absorption by Li_4_SiO_4_ material in a fixed bed reactor at 848 K and 2 MPa, and syngas with the hydrogen concentration of 87% was obtained, which was higher than that obtained when Li_2_ZrO_3_ was used as a CO_2_ acceptor. Essaki et al. [85] introduced commercial Li_4_SiO_4_ pellets into SE-SMR process, and the experiments were carried out on a vertical furnace. It was reported that methane conversion at 550 °C was 80%, and hydrogen concentration reached 93.6 vol.% in syngas. The performance of Li_4_SiO_4_ pellets in the SE-SER process was also tested, and the results showed that the concentrations of hydrogen and CO in syngas were higher than 99 vol.% and less than 0.12 vol.%, respectively, indicating that Li_4_SiO_4_ pellets were promising as the CO_2_ acceptor for the SE-SER process [86]. Zhang et al. [42] reported K_2_CO_3_-doped Li_4_SiO_4_ material coupled well with the Ni/γ-Al_2_O_3_ catalyst, and hydrogen concentration in the syngas was higher than 95 vol.%. In addition, they found that homogeneous distribution of Li_4_SiO_4_ material and catalyst led to higher hydrogen concentration in the syngas.

It can be concluded from the studies above that hydrogen yield and concentration were mainly dependent on the performance of Li_4_SiO_4_ materials, thus Li_4_SiO_4_ materials with superior CO_2_ absorption performance should be investigated. Additionally, Li_4_SiO_4_ materials may be applicable to various sorption-enhanced hydrogen production, and raw materials for gasification could be biomass, sludge, coal, etc.

## 7. Density Functional Theory Studies on Li_4_SiO_4_ Material

Duan et al. [87] studied CO_2_ absorption performance on monoclinic and triclinic phases of Li_4_SiO_4_ using density functional theory, and they found that the thermodynamic properties of the two phases were similar to each other. The calculation results showed that reaction heat of the reaction between Li_4_SiO_4_ and CO_2_ was consistent with the experimental data. Kong et al. [88] reported that the (0 1 0) plane was the most stable low-Miller index plane of Li_4_SiO_4,_ and the adsorption and dissociation behaviors of molecular H_2_O on the Li_4_SiO_4_ (0 1 0) plane were investigated. They found that molecular H_2_O was more inclined to be absorbed on O atoms on the surface.

## 8. Conclusions

Research progress of Li_4_SiO_4_ materials for CO_2_ capture in energy production processes, including hydrogen plants based on sorption-enhanced reforming and fossil fuel-fired power plants, were reviewed in this paper. Thermodynamic and kinetic studies on the preparation and CO_2_ absorption of Li_4_SiO_4_ material were demonstrated, and the diffusion of CO_2_ and ions through the product layer seemed to be the limiting step for CO_2_ absorption by Li_4_SiO_4_ material. Since Li_4_SiO_4_ material prepared by the traditional solid-state reaction method only achieved low CO_2_ absorption capacity, methods to enhance the CO_2_ absorption performance of Li_4_SiO_4_ material were illustrated. Introducing a solid solution and molten salts could reduce the diffusion resistance in the product layer, and using hydration, ball milling, or organic precursors could increase the contact area of CO_2_ and Li_4_SiO_4_, which is beneficial for CO_2_ absorption by Li_4_SiO_4_ material. The sol-gel method seemed to be most appropriate for preparation of Li_4_SiO_4_ material, which is beneficial for the formation of porous structure. The effects of gas contaminants and reaction conditions on CO_2_ absorption performance of Li_4_SiO_4_ material and the applications of Li_4_SiO_4_ material in the sorption-enhanced hydrogen production process were summarized. In view of the current studies reviewed in this work, potential research thoughts and trends are suggested as follows:

(i) Most of the laboratory experiments were carried out on TGA or fixed-bed reactors, while fluidized bed was the common equipment in practical application for the absorption of CO_2_ under most energy production conditions. Additionally, powdery Li_4_SiO_4_ materials could not create effective fluidization, while studies on the performance of pelletized Li_4_SiO_4_ materials were insufficient. As a result, more focus should be attached to the CO_2_ absorption performance of pelletized Li_4_SiO_4_ material in fluidized bed reactors.

(ii) Application of Li_4_SiO_4_ materials on sorption-enhanced hydrogen production is an important aspect, and hydrogen yield and concentration were considerable, while fewer studies involved this area. Additionally, CO_2_ absorption performance of Li_4_SiO_4_ materials in realistic sorption-enhanced hydrogen production conditions (i.e., low CO_2_ concentration in the presence of steam) deserves to be studied.

(iii) Preparation cost of Li_4_SiO_4_ materials is the main problem that limits its industrial application, thus many studies investigated the feasibility of silicon-containing solid wastes as a silicon precursor. However, the main factor that controls the cost of Li_4_SiO_4_ materials is the expensive lithium precursor. As a result, it is suggested that lithium-containing wastes can be tested for the possibility as a lithium precursor, and Li_4_SiO_4_ materials prepared from inexpensive lithium-containing wastes may be promising for large-scale CO_2_ absorption.

## Figures and Tables

**Table 1:** Kinetic parameters of reaction between Li_4_SiO_4_ and CO_2_ for double exponential model [28].

T (°C)	*k*_1_(sec^−1^)	*k*_2_(sec^−1^)	*A*	*B*	*C*
**460**	8.0 × 10^−4^	1.4 × 10^−4^	−3.248	−5.560	108.8
**480**	1.72 × 10^−3^	1.3 × 10^−4^	−3.467	−7.821	111.2
**500**	2.96 × 10^−3^	1.7 × 10^−4^	−3.716	−8.668	112.3
**520**	4.26 × 10^−3^	2.1 × 10^−4^	−4.231	−8.711	112.8
**540**	4.17 × 10^−3^	2.1 × 10^−4^	−4.983	−9.003	113.8
**560**	4.27 × 10^−3^	2.5 × 10^−4^	−9.017	−8.911	116.6

**Table 2:** Summary of CO_2_ absorption performance of Na and K doped Li_4_SiO_4_ materials.

Materials	Molar Ratio of Li/Alkali Metal	Preparation Method	Absorption Conditions	Regeneration Conditions	Cycle No.	Weight Increase (wt.%)	Refs.
SiO_2_, Li_2_CO_3_, K_2_CO_3_	10.5:1	Solid-state reaction	4% CO_2_/N_2_; 580 °C; 60 min	N_2_; 800 °C; 10 min	4	24	[39]
SiO_2_, Li_2_CO_3_, K_2_CO_3_	10.83:1	Solid-state reaction	4% CO_2_/N_2_; 580 °C; 60 min	N_2_; 700 °C; 15 min	25	16	[40]
SiO_2_, Li_2_CO_3_, K_2_CO_3_	17.6:1	Solid-state reaction	CO_2_; 575 °C; 25 min	N_2_; 700 °C; 50 min	10	28	[41]
SiO_2_, Li_2_CO_3_, K_2_CO_3_	17.6:1	Solid-state reaction	CO_2_; 650 °C; 15 min	90% H_2_O/N_2_; 650 °C; 15 min	22	21	[42]
SiO_2_, CH_3_COOLi, K_2_CO_3_	43.7:1	Impregnated suspension	15% CO_2_/N_2_; 550 °C; 30 min	N_2_; 750 °C; 10 min	40	315	[43]
SiO_2_, CH_3_COONa, Na_2_CO_3_	16:1	Impregnated suspension	15% CO_2_/N_2_; 550 °C; 30 min	N_2_; 750 °C; 10 min	40	28	[43]
SiO_2_, Li_2_CO_3_, Na_2_CO_3_	49:1	Solid state	CO_2_; 700 °C; 30 min	Air; 900 °C; 30 min	5	32	[44]
SiO_2_, LiNO_3_, NaF	41:1	Template	15% CO_2_/N_2_; 600 °C; 35 min	N_2_; 700 °C; 20 min	10	31	[45]
SiO_2_, LiNO_3_, NaCl	133:1	Solid state with hydration	15% CO_2_/N_2_; 575 °C; 40 min	N_2_; 700 °C; 10 min	10	32	[46]

**Table 3:** CO_2_ absorption performances of Li_4_SiO_4_ materials with different molar ratios of Li_2_CO_3_ to SiO_2_ [65].

Molar Ratio	2.0:1	2.1:1	2.2:1	2.3:1	2.4:1	2.6:1	2.8:1
Weight gain (%)	116	122	124	129	129	130	116
